# Irreversible EGFR Inhibitor EKB-569 Targets Low-LET γ-Radiation-Triggered Rel Orchestration and Potentiates Cell Death in Squamous Cell Carcinoma

**DOI:** 10.1371/journal.pone.0029705

**Published:** 2011-12-29

**Authors:** Natarajan Aravindan, Charles R. Thomas, Sheeja Aravindan, Aswathi S. Mohan, Jamunarani Veeraraghavan, Mohan Natarajan

**Affiliations:** 1 Department of Otolaryngology, Head and Neck Surgery, University of Texas Health Science Center at San Antonio, San Antonio, Texas, United States of America; 2 Department of Radiation Oncology, The University of Oklahoma Health Sciences Center, Oklahoma City, Oklahoma, United States of America; 3 Department of Radiation Medicine, Oregon Health and Science University Knight Cancer Institute, Portland, Oregon, United States of America; 4 Department of Pathology, The University of Oklahoma Health Sciences Center, Oklahoma City, Oklahoma, United States of America; 5 Department of Pediatrics, The University of Oklahoma Health Sciences Center, Oklahoma City, Oklahoma, United States of America; Ottawa Hospital Research Institute, Canada

## Abstract

EKB-569 (Pelitinib), an irreversible EGFR tyrosine kinase inhibitor has shown potential therapeutic efficiency in solid tumors. However, cell-killing potential in combination with radiotherapy and its underlying molecular orchestration remain to be explored. The objective of this study was to determine the effect of EKB-569 on ionizing radiation (IR)-associated NFκB-dependent cell death. SCC-4 and SCC-9 cells exposed to IR (2Gy) with and without EKB-569 treatment were analyzed for transactivation of 88 NFκB pathway molecules, NFκB DNA-binding activity, translation of the NFκB downstream mediators, Birc1, 2 and 5, cell viability, metabolic activity and apoptosis. Selective targeting of IR-induced NFκB by EKB-569 and its influence on cell-fate were assessed by overexpressing (p50/p65) and silencing (ΔIκBα) NFκB. QPCR profiling after IR exposure revealed a significant induction of 74 NFκB signal transduction molecules. Of those, 72 were suppressed with EKB-569. EMSA revealed a dose dependent inhibition of NFκB by EKB-569. More importantly, EKB-569 inhibited IR-induced NFκB in a dose-dependent manner, and this inhibition was sustained up to at least 72 h. Immunoblotting revealed a significant suppression of IR-induced Birc1, 2 and 5 by EKB-569. We observed a dose-dependent inhibition of cell viability, metabolic activity and apoptosis with EKB-569. EKB-569 significantly enhanced IR-induced cell death and apoptosis. Blocking NFκB improved IR-induced cell death. Conversely, NFκB overexpression negates EKB-569 -induced cell-killing. Together, these pre-clinical data suggest that EKB-569 is a radiosensitizer of squamous cell carcinoma and may mechanistically involve selective targeting of IR-induced NFκB-dependent survival signaling. Further pre-clinical *in-vivo* studies are warranted.

## Introduction

Head and neck squamous cell carcinoma (HNSCC) is the sixth most common cancer in the world and accounts for 90% of malignant neoplasias of the upper respiratory system [1]. Despite recent advances in the management of locally advanced HNSCC, the overall survival of patients has improved only marginally over the past three decades [2] mainly due to development of therapy-induced chemo and radioresistance. To that note, in recent years there has been substantial interest in developing novel therapeutic agents that specifically target growth factor pathways that, are dysregulated in tumor cells. Such targeted “biological” agents might offer alternative treatment options for patients refractive to chemoradiotherapy. Also, with unique mechanisms of action and toxic profiles that generally do not overlap, targeted agents and standard therapies can be used in combinations to enhance overall treatment efficacies and prevent dose reduction.

Because many solid tumors, including HNSCC have hyper activated epidermal growth factor receptor (EGFR) [3],[4], there has been great interest in the use of EGFR inhibitors to control cancer growth. EGFR is a 170 kDa glycoprotein containing an extracellular ligand binding domain, and an intracellular tyrosine kinase (TK) domain [5]. Upon binding to ligands such as EGF or TGFα, EGFR dimerizes with itself (homodimers) or other members of the family such as c-ErbB-2 (heterodimers). Upon dimerization, TK activation increases and receptor gets autophosphorylated at tyrosine residues. Phosphorylated EGFR (p-EGFR), like other activated receptor TKs, involved in phosphorylation and activation of several signal transduction pathways including phosphoinositide 3-kinase-AKT, extra cellular signal-regulated kinase 1and 2 (ERK1/2), and the signal transducer and activator of transcription 3 (STAT3). Activation of these signal transduction pathways subsequently activate key transcriptional machineries such as NFκB that promote tumor growth and progression by inducing inhibition of apoptosis, proliferation, maturation,clonal expansion, invasion, and metastasis.

NFκB is a member of the c-*rel* proto-oncogene family found within the promoter and enhancer region of a wide variety of genes involved in proliferation, cell cycle control [6], [7], oncogenic activation [8], cell growth, differentiation and metastasis [9], [10]. NFκB is retained in the cytoplasm by association with the inhibitory protein IκB. On phosphorylation, IκB is ubiquitinated and subsequently degraded by the 26S proteasome, resulting in the liberation of NFκB. NFκB can then enter into the nucleus to regulate the expression of downstream genes. Elevated NFκB activity has been linked with tumor resistance to chemotherapy and IR [11] in a number of cancer types, including head and neck cancer [12]. Conversely, inhibition of NFκB favors pro-apoptotic processes, decreases growth and clonogenic survival [13]–[15] and enhances chemo/radiosensitivity [16]–[20]. In addition to this persistant activation of growth-promoting signaling pathways, development of HNSCC also involves the accumulation of genetic and epigenetic alterations in tumor-suppressor proteins.. The activation of EGFR is a frequent event in HNSCC, and has provided the molecular basis for current efforts aimed at evaluating the clinical activity of EGFR inhibitors in HNSCC [21], [22]. However, to date, the role of EGFR-dependent NFκB in the functional orchestration of HNSCC progression and metastasis is poorly realized [22], [23]. Since NFκB is able to regulate more than 150 genes, and is able to functionally orchestrate many steps in carcinogenesis, tumor progression and metastasis, it is important to delineate the efficacy of potential EGFR-TK inhibitors that target the NFκB-dependent HNSCC cell survival advantage.

The two most commonly employed strategies in drug development are introducing covalent (irreversible) binding of the drug target and and broadening the affected receptor tyrosine kinase targets of the drug within the cell. Currently, the second generation of EGFR TKI compounds is emerging from the drug developmental pipeline and being introduced into clinical trials. Many of these second-generation compounds form tighter covalent bonds with their target, which should theoretically increase their effectiveness by prolonging the inhibition of EGFR signaling to the entire lifespan of the drug-bound receptor molecule. In cell culture systems, such irreversibly binding TKIs can effectively kill cells that have acquired resistance to first-generation TKIs [24]. As per the other common theme of drug development, second-generation EGFR TKI have been developed that, in addition to blocking EGFR signaling, target multiple kinases in the ErbB family. The signaling network that emerges from the ErbB family of transmembrane TK receptors (of which EGFR is a member) is large, interconnected, and redundant, with many possible routes between the ligand at the cell surface and the message destination within the nucleus [25]. It is this diversity in possible signal transduction routes that allows a cell to have flexibility and, in the case of cancer cells treated with anticancer agents, facilitates resistant cell clones that bypass the inhibited receptor [26]. Blocking multiple signaling pathways with either a combination of agents or a single but multi-targeted agent has been synergistic in its effects in preclinical models [27]. Second-generation EGFR TKIs have been developed that target additional members of the ErbB family or ‘***other downstream or parallel pathways such as the NFκB pathway***’. EKB-569 (Pelitinib; WAY-172569), a 4-Dimethylamino-but-2-enoic acid [4-(3-chloro-4-flurophenylamino)-3-cyano-7ethoxy-quinolin-6-yl]-amide is one such second generation irreversibly-binding inhibitor of EGFR TK activity [28]. In this study, we examined the efficacy of EKB-569 in inhibiting ionizing radiation (IR)-induced NFκB activity, in modulating the transcription of 88 NFκB-dependent signal transduction molecules, in activating translation of NFκB-mediated downstream Birc1, 2 and 5 protein, in reducing cell viability, and metabolic activity and apoptosis. Further, we delineated the selective targeting of IR-induced NFκB through EKB-569 and its direct influence in HNSCC cell-fate.

## Materials and Methods

### Cell Culture

Human tongue squamous cell carcinoma SCC-4 and SCC-9 cells were obtained from ATCC (Manassas, VA) and maintained as monolayer cultures in DMEM/F-12 50/50 (Mediatech Inc., Herndon, VA) growth medium supplemented with 1.5 g/L sodium bicarbonate, 2 mM L-glutamine, 15 mM HEPES, 1% NEAA, 1% MEM vitamins, 5000 I.U/ml penicillin/5000 µg/ml streptomycin, 1% sodium pyruvate, and 10% FBS (Invitrogen, Carlsbad, CA). For passage and for all experiments, the cells were detached using trypsin (0.25%)/EDTA (1%), resuspended in complete medium, counted (Countess, Invitrogen) and incubated in a 95% air/5% CO2 humidified incubator.

### Irradiation experiments

SCC-4 and SCC-9 cells were exposed to 2Gy using Gamma Cell 40 Exactor (Nordion International Inc, Ontario, Canada) at a dose rate of 0.81Gy/min. Irradiated cells were examined for IR-induced alterations in NFκB signal transduction, selective yet, sustained NFκB activity, NFκB's role in survival advantage and to identify the efficacy of EKB-569 on IR-induced NFκB dependent HNSCC progression. Mock irradiated cells were treated identical except that the cells were not subjected to IR. Irradiated cells were incubated at 37°C for additional 1, 3, 6, 24, 48 and 72 h. All experiments were repeated at least three times in each group.

### Plasmid preparation and DNA Transfection

Transient transfection of NFκB p65 and p50 subunits was carried out by the lipofection method using Effectene™ reagent (Qiagen, Inc., Valencia, CA) as described in our earlier studies [29]. NFκB inhibition was achieved using transient transfection of S32A/S36A double mutant IκBα (ΔIκBα, Upstate biotechnology, Lake Placid, NY) as reported in our earlier studies [29] . The mutated form of IκBα with a serine-to-alanine mutation at residues 32 and 36 does not undergo signal-induced phosphorylation and thus remains bound to NFκB subsequently preventing nuclear translocation and DNA binding. After 18 h, transfection medium was replaced with growth medium before IR.

### Electrophoretic Mobility Shift Assay (EMSA)

Nuclear protein extraction and electrophoretic mobility shift assay for NFκB, AP-1 and SP-1 were performed as described in our earlier studies [29]. Autoradiograms were overexposed in order to reveal the low inhibitory effects that were below the constitutive level. Densitometry analysis was performed using a BioRad Multi-Analyst software package with an integrated density program. Group-wise comparisons were made using ANOVA with Tukey's post-hoc correction. A P value of <0.05 is considered statistically significant. For the competition assay, the nuclear extract was pre-incubated with unlabeled homologous NFκB oligonucleotide followed by addition of [γ-^32^P]-ATP labeled NFκB probe. Supershift analysis was performed as described earlier [29].

### Immunoblotting

Total protein extraction and immunoblotting were performed as described in our earlier studies [29]. Rabbit polyclonal anti-IκBα, Birc1, 2, 5 or Bax antibody (Santa Cruz) were used to detect the respective protein expression levels between the EKB treated, IR exposed and control groups. Blots were stripped and reprobed with mouse monoclonal anti-α-tubulin antibody (Santa Cruz) to determine equal loading of the samples. One diamentional gel analysis was performed using a BioRad Multi-Analyst software package with an integrated density program. Group-wise comparisons were made using ANOVA with Tukey's post-hoc correction. A P value of <0.05 is considered as statistically significant.

### Real-Time QPCR profiling of NFκB signaling pathway molecules

Total RNA extraction and real-time QPCR profiling were performed as described in our earlier studies [29] . We used human NFκB signaling pathway profiler (Realtimeprimers.com, Elkins Park, PA) containing 88 genes representing 8 functional groups including (i) Rel/NFκB/IκB family, (ii) NFκB responsive genes, (iii) Ligands & Transmembrane receptors, (iv) Adaptor proteins, (v) Signal transduction kinases, (vi) Transcription factors, (vii) Cell death/survival molecules, and (viii) Other factors. We started with this highly selected QPCR profiler instead of an all-encompassing gene array because the selected genes entail a well-characterized profile governing NFκB signal transduction and transcriptional targets, hence facilitating interpretation of data, simplifying data acquisition and analysis, and avoiding genes not functionally characterized. Furthermore, QPCR profiling allows detection and quantification of gene expression in real-time. Each profiling plate was also equipped with reverse transcription control, positive PCR control, genomic DNA control and five housekeeping genes – *β-Actin*, *GAPDH*, *Rpl13a*, *HPRT1* and *β2M*. The ΔΔ^ct^ values were calculated by normalizing the gene expression levels to the expression of the housekeeping genes. The normalized data were then compared between groups, and the relative expression level of each gene was expressed as fold change. When comparing each gene's signal intensity between groups, we used a twofold or more (≥2 fold) increase or decrease to represent “stringent” criteria for upregulation or downregulation and an increase/decrease of <2 fold to represent “less stringent” criteria. Classifying gene regulation criteria in this manner can provide an index of reliability of the gene expression data [29].

### Cell Viability

Trypan blue dye exclusion assay was used to identify IR modulated cell viability in HNSCC cells and further, to determine the efficacy of EKB-569 in this setting. Cells exposed to IR alone and cells pre-treated with EKB-569 followed by exposure to IR, were sequentially analyzed with the Countess automated cell counter (Carlsbad, CA). Furthermore, to determine the efficiency of EKB-569 in targeting IR-induced NFκB dependent cell viability, trypan blue exclusion assay was performed in NFκB over-expressed HNSCC cells exposed to EKB-569. Group-wise comparisons were made using ANOVA with Tukey's post-hoc correction. A P value of <0.05 is considered statistically significant.

### Cell survival by MTT assay

Cell survival was analyzed using MTT assay as described in our previous studies [29]. HNSCC cells at a density of 1000 cells/300 µl in a 24-well plate were either (i) mock-irradiated, (ii) exposed to IR alone, (iii) treated with EKB-569 (0.5, 1.0, 2.0 and 5.0 µg) alone, (iv) pretreated with EKB-569 (5.0 µg) followed by exposure to IR, (v) prior transfection with ΔIκBα followed by exposure to IR, or (vi) prior transfection with p50/p65 treated with or without EKB-569. The treated and/or exposed cells were added with 3-(4,5-dimethyl-2-thiazolyl)-2,5-diphenyl-2H-tetrazolium bromide (30 µL/well from 5 mg/mL stock) for 4 h after 24, 48 and 72 h of post-IR. Solubilization of converted purple formazan dye was accomplished by acid-isopropanol with continuous shaking at 37°C. The reaction product was quantified by measuring the absorbance at 570 nm using Synergy II micro plate reader (Biotek). Cell survival response was compared using ANOVA with Tukey's post-hoc correction.

### Nuclear morphology by dual staining

SCC-4 cells (5×10^5^ cells in 500 µl of complete growth medium) grown in 4-well plate (Nunc) were either: 1) sham treated, 2) treated with EKB-569 (0.5–5.0 µg), 3) exposed to IR with or without prior EKB-569/ΔIκBα transfection, or 4) transfected with p50/p65 subunit with or without prior EKB-569 treatment. The cells were analyzed for nuclear morphology as described earlier [30]. In brief, the medium was replaced with a fresh medium containing reduced serum (2%) without any added growth factors and incubated further for 16 h at 37°C in air/CO2 incubator. The cells were then stained with acridine orange (1 µg/ml) and ethidium bromide (1 µg/ml) and immediately examined for the morphological characteristics of apoptosis at 200× magnification using an Olympus VANOX fluorescent microscope. Four morphological states were examined: (1) viable cells with normal nuclei (bright green chromatin with organized structure); (2) viable cells with apoptotic nuclei (green chromatin which are highly condensed and/or fragmented); (3) non-viable cells with normal nuclei (bright orange chromatin with organized structure); and (4) non-viable cells with apoptotic nuclei (bright orange chromatin which is highly condensed or fragmented).

## Results

### EKB-569 selectively inhibits IR-induced persistent activation of NFκB

**Figure 1:**
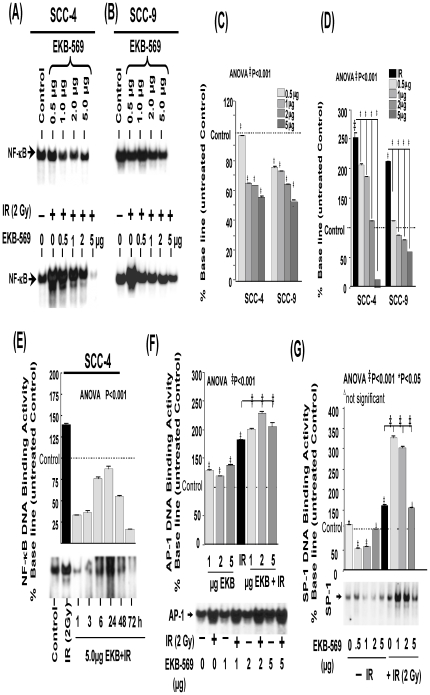
Effect of EKB-569 on radiation modulated NFκB, AP1 and SP1 DNA binding activity. A representative autoradiograms showing the NFκB-DNA binding activity in the nuclear extracts of human SCC-4 cells (**A**) or SCC-9 cells (**B**) that are either treated with EKB-569 alone (upper panel) or in combination with IR (Lower panel). NF-kB-specific bands are indicated by an arrow head. Autoradiogram was slightly overexposed to reveal EKB-inhibited NF-kB-specific bands. Densitometric analysis of three independent experiments showing dose-dependent inhibition of NFκB-DNA binding activity in SCC-4 cells (C) and SCC-9 cells (**D**). (**E**) Time-dependent inhibition of NFκB-DNA binding activity in human SCC-4 cells by EKB-569 (5.0 µg) in the presence or absence of IR exposure. EMSA was carried out in the nuclear extract at 1, 3, 6, 24, 48 and 72 h post-exposure. (**F**) Representative autoradiogram from three independent experiments showing AP-1 DNA binding activity in SCC-4 cells treated with EKB-569 (1.0, 2.0 and 5.0 µg) or exposed to IR in the presence or absence of EKB-569. (**G**) Representative autoradiogram from three independent experiments showing SP-1 DNA binding activity in SCC-4 cells treated with EKB-569 (0.5, 1.0, 2.0 and 5.0 µg) or exposed to IR in the presence or absence of EKB-569.

The effect of EKB-569 in selectively inhibiting IR-induced NFκB-DNA binding activity was elucidated using four different approaches. First, we investigated whether EKB-569 as a stand-alone compound, could modulate NFκB activity in both SCC-4 and SCC-9 HNSCC cells. Compared to untreated cells, EKB-569 treatment dose-dependently inhibited NFκB DNA binding activity with a substantial inhibition at 5.0 µg (Fig. 1A & B). Next, to unveil the radiosensitizing efficacy of EKB-569, HNSCC cells mock-irradiated, exposed to IR or treated with EKB-569 (0.5, 1.0, 2.0 or 5.0 µg) and then exposed to IR were analyzed for alterations in NFκB activity. Unlike the mock-IR controls, IR at 2 Gy significantly (P<0.001) induced NFκB-DNA binding activity in both SCC-4 and SCC-9 cells (Figure 1 A & B, bottom panel). This IR-induced NFκB activity was drastically (P<0.001) inhibited with EKB-569 treatment in a dose dependent manner (Fig. 1D) in both cell types. It is interesting to note that at 5.0 µg concentration, EKB-569 completely suppressed IR-induced NFκB activity even below the constitutive (mock-IR) levels in this setting. Further, to delineate whether EKB-569 persistently inhibits IR-induced NFκB or there is recovery of IR-induced NF-kB activity over time, SCC-4 cells pretreated with EKB-569 and exposed to IR were examined for 3 days post-radiation exposure. EKB-569-induced inhibition of IR-induced NFκB DNA-binding activity remained at the same decreased level at all time points investigated (Figure 1E). Densitometric analysis revealed a significant (P<0.001) inhibition of IR-induced NFκB DNA-binding activity up to at least 3 days post-radiation exposure. (Fig. 1F). To confirm the specificity of the EMSA band seen in Figure 1 A and B, a competition binding assay was performed. The NFκB DNA-binding activity was competitively reduced to 47% and 36.4% by the addition of 0.02 and 0.2 pmol of homologous unlabeled NFκB specific-double stranded oligonucleotide, respectively. Supershift analysis with p50 and p65 antibodies further confirmed that the gel shifted bands are indeed NFκB (data not shown).

**Figure 2:**
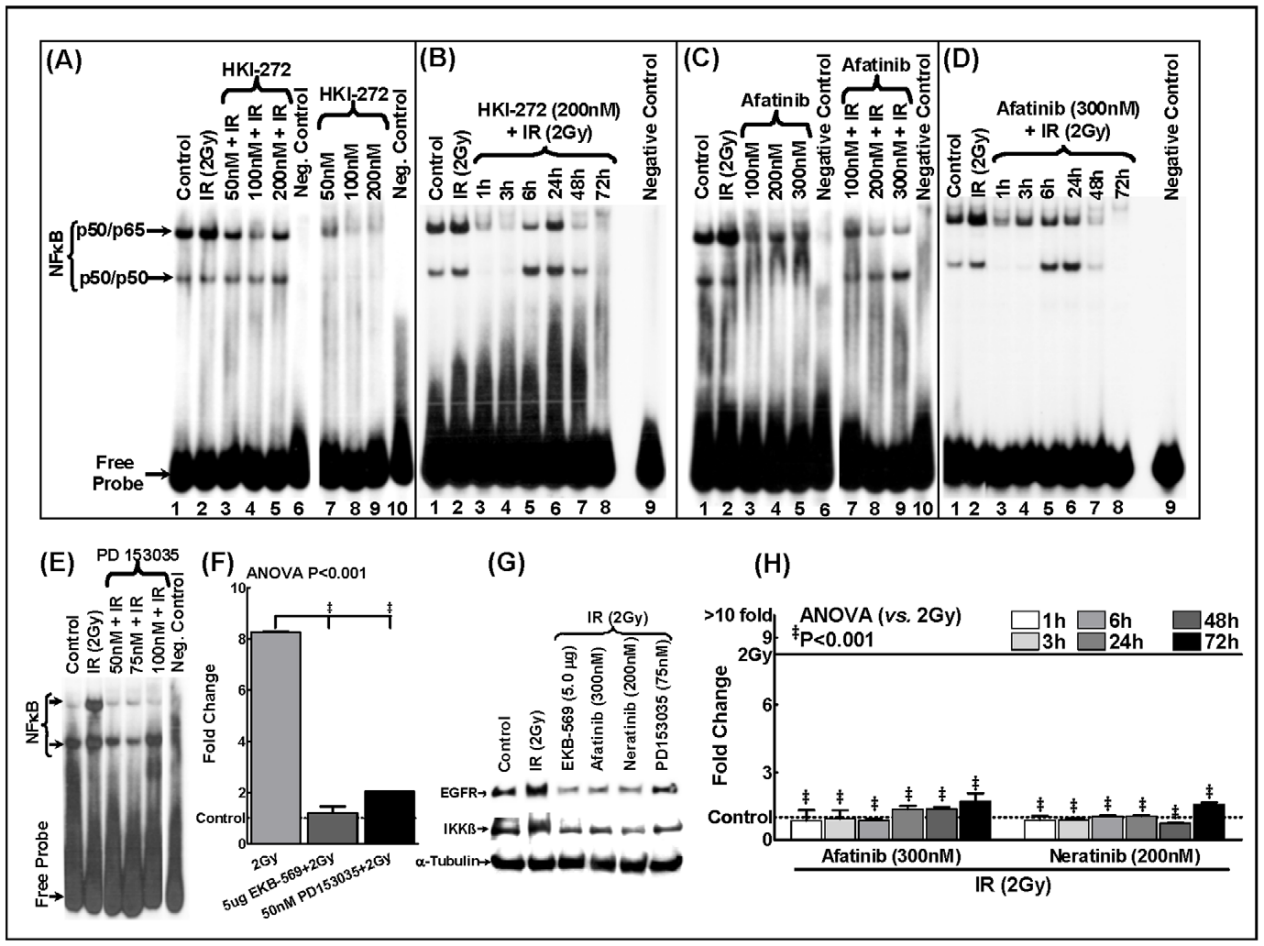
Effect of EGFR inhibitors on NFκB DNA binding activity, EGFR mRNA and, EGFR and IKKβ protein levels. (**A**) Representative autoradiogram showing the NFκB-DNA binding activity in the nuclear extracts of human SCC-4 cells exposed to IR (2Gy) or treated with 50, 100 or 200 nM HKI-272 (neratinib) prior to IR exposure. Neratinib treatment significantly inhibited IR-induced NFκB DNA binding activity (Left panel). Representative autoradiogram showing the NFκB-DNA binding activity in human SCC-4 cells exposed to 50, 100 or 200 nM neratinib (Right panel). Compared to the mock-IR cells, neratinib induced a dose-dependent suppression of NFκB activity in these cells. (**B**) Representative autoradiogram showing the NFκB-DNA binding activity in human SCC-4 cells exposed to IR with or without Neratinib (200 nM) and harvested after 1, 3, 6, 24, 48 and 72 h. Neratinib persistently inhibited IR-induced NFκB-DNA binding activity at all time points investigated. (**C**) Representative autoradiogram showing the NFκB-DNA binding activity in human SCC-4 cells exposed to 100, 200 or 300 nM afatinib. Compared to the mock-IR cells, afatinib induced a dose-dependent suppression of NFκB activity (Left panel). Representative autoradiogram showing the NFκB-DNA binding activity in SCC-4 cells exposed to IR or treated with 100, 200 or 300 nM afatinib and exposed to IR. Afatinib treatment significantly inhibited IR-induced NFκB DNA binding activity (Right panel). (**D**) Representative autoradiogram showing the NFκB-DNA binding activity in human SCC-4 cells exposed to IR with or without afatinib (300 nM) and harvested after 1, 3, 6, 24, 48 and 72 h. Afatinib treatment persistently inhibited IR-induced NFκB-DNA binding activity at all time points investigated. (**E**) Representative autoradiogram showing the NFκB-DNA binding activity in human SCC-4 cells exposed to IR or treated with 50, 75 or 100 nM PD 153035 hydrochloride (a potent EGFR-TK inhibitor) and exposed to IR. PD153035 treatment induced a significant dose-dependent inhibition of IR-induced NFκB DNA binding activity. (**F**) Real-time QPCR analysis showing EGFR mRNA levels in SCC-4 cells mock-irradiated, exposed to 2Gy and in cells treated either with EKB-569 (5.0 µg) or PD153035 (50 nM) and exposed to IR. (**G**) Immunoblot showing complete suppression of radiation induced EGFR and IKKβ levels in SCC-4 cells pretreated with EKB-569 (5.0 µg), afatinib (300 nM), neratinib (200 nM) or PD153035 (75 nM). (**H**) QPCR analysis showing complete and sustained (up to 72 h) suppression of radiation induced EGFR transcriptional levels in SCC-4 cells treated with either afatinib (300 nM) or neratinib (200 nM).

Next, to demonstrate that the inhibition of NFκB signaling pathway is not a EKB-569 compound-specific effect and that the proposed combination (IR and EGFR inhibition) can be carried out on to the clinic with any other EGFR compound, we incubated the SCC-4 cells with other commonly used irreversible EGFR blockers, afatinib and neratinib (HKI-272). Afatinib and neratinib dose-dependently inhibit NF-kB DNA-binding activity (Figure 2 A, B, C & D). The inhibition of NFκB was found to be persistent up to at least 72 h (Figure 2H). To further validate whether EKB-569 directly inhibits NFκB activity, we examined the inhibitory effect on the activity of unpstream kinases. Data presented in Figure 2G shows EKB-569 and other related EGFR inhibitors, afatinib (300 nM) and neratinib (200 nM) significantly block the expression of IR-induced upstream IκB kinase beta (IKK-β). Additionally, we confirmed that EKB-569-mediated inhibition of NF-κB is EGFR-dependent. EGFR-knockdown experiments with a widely used specific EGFR inhibitor, PD153035 were performed to confirm the EGFR-mediated NFκB inhibition. Cells incubated with PD153035 at concentrations 50, 75 and 100 nM clearly showed a significant decrease in radiation-induced NFκB DNA binding activity and mRNA expression similar to the cells incubated with EKB-569 (Figure 2 E&F).

In order to determine whether EKB-569 selectively targets NFκB or the global transcription machinery in general, we analyzed the effect of EKB-569 on IR-modulated AP-1 and SP-1 transcription factors. SCC-4 cells mock-irradiated, treated with EKB-569 (0.5–5.0 µg), exposed to IR or, treated with EKB-569 (0.5–5.0 µg) and then exposed to IR were examined for AP-1 and SP-1 DNA binding activity (Figure 1 F&G). In contrast to the NF-kB pathway response, EKB-569 by itself, without radiation exposure, fails to inhibit the constitutive levels of AP-1 DNA-binding activity. On the other hand, with regard to SP-1, EKB-569 inhibits its activity at the lower concentrations of 1 and 2 ug, but not at the higher (5 ug) concentration. More interestingly, with the addition of EKB-569 further increased the activation of AP-1 and the SP-1 induced by IR exposure. These results confirmed that the mechanism of EKB-569-mediated radiosensitization is acting specifically through NF-kB pathway.

### EKB-569 inhibits IR-induced transcriptional modulation of NFκB signal transduction and pathway molecules in HNSCC cells

**Figure 3:**
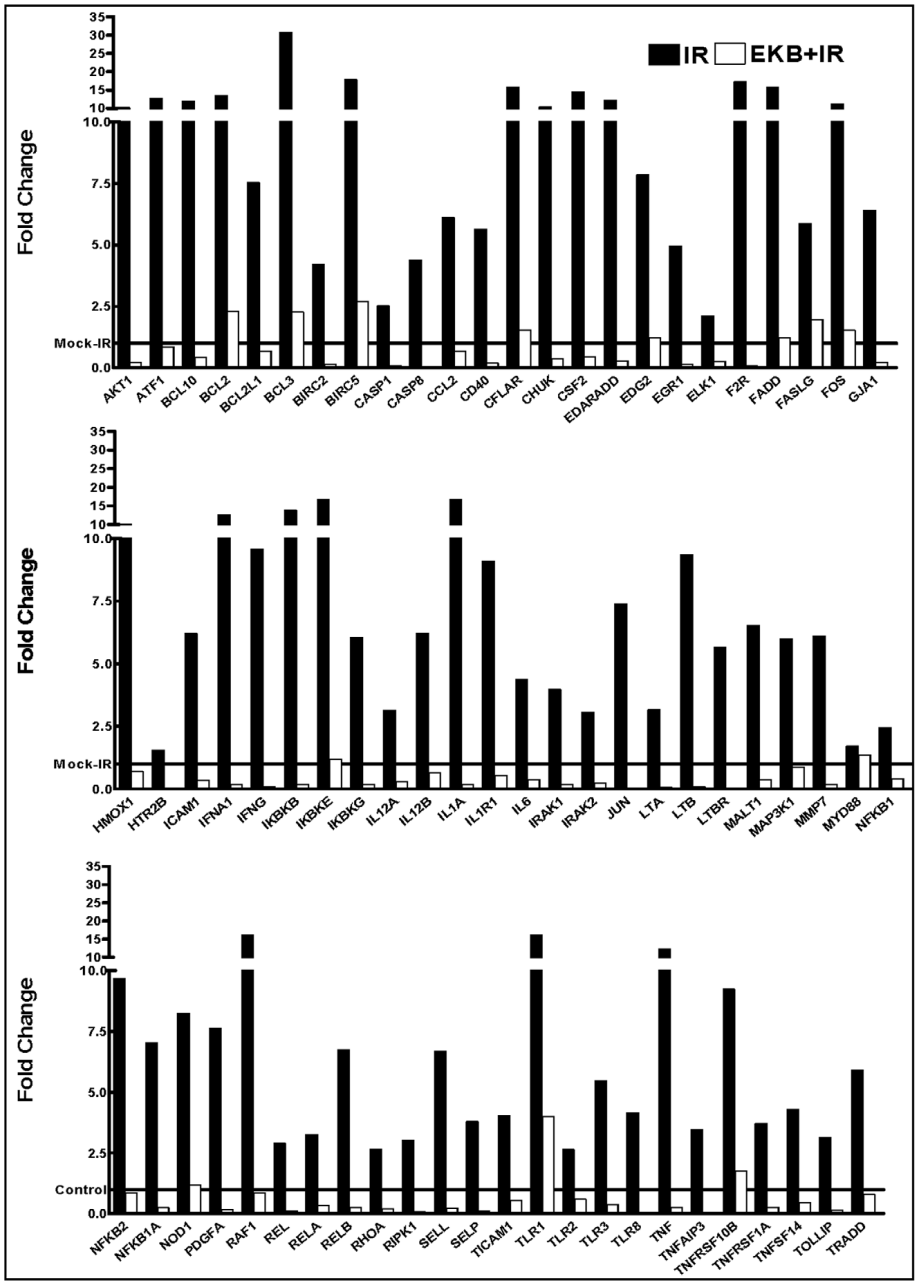
Real time QPCR profiling: Histograms showing IR-induced NFκB-dependent downstream signal transduction molecules and the effect of EKB-569 (5.0 µg) on these IR-modulated genes in human SCC-4 cells.

To further to substantiate our findings of IR-induced NFκB activation and EKB-569 associated selective targeting, SCC-4 cells mock-irradiated, exposed to IR or pretreated with EKB-569 (5.0 µg) and then exposed to IR were examined for transcriptional changes in 88 NFκB signal transduction and downstream target genes (Figure S1). Compared to mock-IR controls, IR exposure upregulated 74 genes, down regulated two genes, while having no effect on the expression of 12 genes. Though, originally we intended to classify the gene expression implying less stringent (overall) and stringent (≥2 fold) criteria, there is only one gene, *Myd88* showed less than 2 fold (1.4) while remaining 73 genes showed significant (≥2 fold) upregulation compared to untreated control. Conversely, EKB-569 pre-treatment profoundly inhibited 72 of 74 IR-induced genes in this setting (Figure 3). Interestingly, expression of two genes, *TLR4* and *Ppm1A* were significantly increased with EKB-569. A plethora of scientific literature demonstrates the functional significance of these NFκB-dependent signaling and target molecules in tumor cell radioresistance suggesting that inhibitory approaches of these molecules may benefit radiosensitization.

### EKB-569 regulates NFκB dependent downstream Birc 1, 2 and 5 and upregulates pro-apoptotic Bax in HNSCC cells

**Figure 4:**
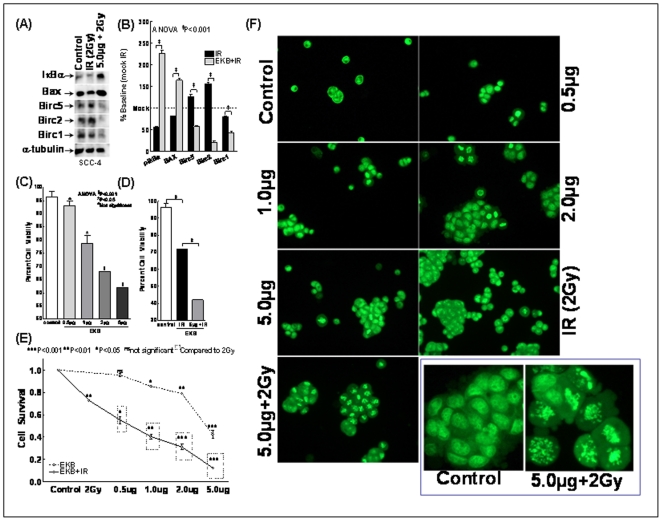
Effect of EKB-569 on radiation modulated prosurvival signaling molecules, cell viability, survival and/or death. (**A**) Representative immunoblot showing expression levels of IκBα, pro-apoptotic Bax and anti-apoptotic Birc1, 2 and 5 in human SCC-4 cells exposed to IR or treated with EKB-569 (5.0 µg) prior to IR exposure. α-tubulin was used to show equal loading of protein samples. (**B**) Semi-quantitative 1D gel analysis showing increased IκBα and Bax levels in EKB-569 treated cells. EKB-569 treatment significantly suppressed Birc1, 2 and 5 in these IR-exposed SCC-4 cells. (**C**) Histograms showing the percent cell viability in cells treated with EKB-569 (0.5, 1.0, 2.0 and 5.0 µg). EKB-569 inflicted a dose dependent inhibition of cell viability in this setting. (**D**) Histograms showing the percent cell viability in cells either mock-irradiated, exposed to IRexposure or treated with EKB-569 (5.0 µg) and exposed to IR. Compared to the mock-IR cells, IR resulted in reduced cell viability. Relatively, EKB-569 treatment significantly conferred IR-inhibited cell viability. Cell viability was measured using Trypan-blue dye exclusion assay and counted in automated countess cell counter. (**E**) Cell survival in mock-IR, EKB-569 (0.5, 1.0, 2.0 and 5.0 µg) treated and in irradiated cells with or without EKB-569 treatment. MTT assay was used to analyze the induced cytotoxicity and the reaction product was quantified by measuring the absorbance at 570 nm. Percent cell survival was calculated as (mean of test wells/mean of control wells) ×100 and compared using ANOVA. EKB-569 induced a dose dependent inhibition of cell survival. Like-wise IR suppressed cell survival and this IR-inhibited cell survival was further inhibited with EKB-569 in a dose dependent fashion. (**F**) Nuclear morphology with dual staining showing apoptotic characteristics in cells either mock-IR, treated with EKB-569 (0.5, 1.0, 2.0, 5.0 µg), exposed to IR, or treated with EKB-569 and exposed to IR. ***Insert:*** High magnification photomicrographs showing chromatin with organized structures indicating viable cells with normal nuclei in untreated control cells and, chromatin with blebbing, nuclear condensation, and fragmentation indicating typical apoptotic characteristics in cells treated with 5.0 µg of EKB-569 and exposed to IR.

QPCR profiling demonstrated a significant inhibition of IR-induced NFκB-dependent downstream pro-survival protein, Birc 2 and 5 upon EKB-569 treatment (Figure 3). In order to confirm the IR-induced modulations and to validate the functional significance of EKB-569-mediated regulation, we investigated whether the transcriptional machinery modulation is in fact translated to the protein level. First, immunoblotting analysis confirmed the involvement of post-translational modification of IκB in IR-induced NFκB. Further, we observed a significant setback of IR-inhibited IκBα levels upon EKB-569 treatment. This correlated well with induced NFκB activity data (Figure 1 A–D). Compared to mock-IR controls, we observed a significant induction of BIRC 2 and 5 levels (Figure 4 A&B) reflecting and correlating well with their mRNA expression levels. More importantly, treatment with EKB-569 completely (P<0.001) inhibited IR-induced BIRC2 and 5 in SCC-4 cells. Though IR did not show induced expression of BIRC 1 in this setting, we observed a conferring inhibition of this protein with EKB-569. Conversely, we observed a significant induction of pro-apoptotic Bax in cells pre-treated with EKB-569.

### EKB-569 confers radiosensitization in HNSCC cells

To identify the efficacy of EKB-569 at the cellular or tissue level of HNSCC radiosensitization, we examined their potential in conferring functional endpoints like cell viability, survival and apoptotic death. First, trypan blue exclusion assay demonstrated that EKB-569 as a stand-alone compound induced dose-dependent inhibition of SCC-4 cell viability with a maximum (P<0.001) inhibition at 5.0 µg concentration (Figure 4C). Similarly, unlike the mock-irradiated control, cells exposed IR significantly (P<0.001) inhibited HNSCC cell viability (Figure 4D). More importantly, compared to IR exposed cells, EKB-569 (5.0 µg) treatment significantly (P<0.001) conferred IR-inhibited cell viability. Substantiating our cell viability data, MTT analysis revealed a dose dependent inhibition of metabolic activity with EKB-569 treatment (Figure 4E). To that end, at low concentration (0.5 µg) we did not see any significant inhibition of cell survival. However, with increase in EKB-569 concentration we observed a significant (1.0 µg, P<0.05; 2.0 µg, P<0.01 and 5.0 µg, P<0.001) inhibition of cell survival in this setting. On the other hand, compared to mock-irradsiated, cell exposed to IR showed significant (P<0.01) suppression of cell survival (Figure 4E). Addition of EKB-569 significantly conferred IR-inhibited cell survival in a dose dependent fashion. Even concentrations as low as 0.5 µg significantly conferred IR-induced cell death and we observed a complete inhibition of cell survival in IR-exposed cells with 5.0 µg demonstrating the radiosensitizing potential of EKB-569 in HNSCC cells. Further, nuclear morphology with dual staining showed bright green chromatin with organized structures in untreated control cells indicating viable cells with normal nuclei (Figure 4F). Where as, cells treated with EKB-569 showed typical apoptotic features of bright orange chromatin with blebbing, nuclear condensation, and fragmentation. We observed a dose dependent increase in apoptosis after 0.5, 1.0, 2.0 and 5.0 µg of EKB-569. Consistent with our cell viability and survival data, we observed an induced cell death in cells exposed to IR with bright orange chromatin with blebbing, nuclear condensation, and fragmentation. More importantly, compared to IR alone, cells pre-treated with EKB-569 (5.0 µg) and exposed to IR showed extensive apoptotic characteristics and demonstrated a radiosensitizing potential in HNSCC cells (Figure 4F).

### EKB-569 targets IR-induced NFκB- regulated radiosensitization

**Figure 5:**
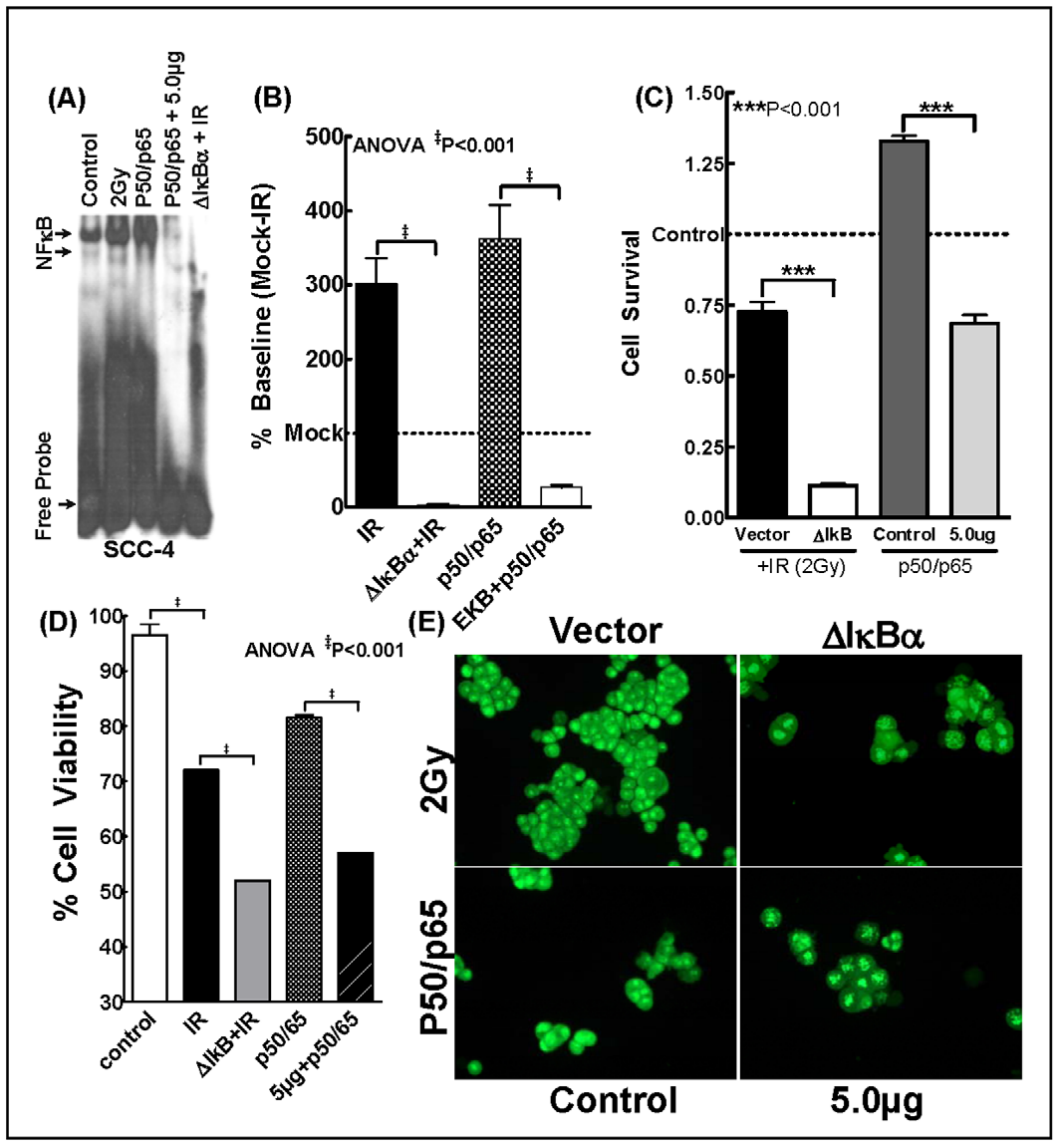
IR-induced NFκB regulates radioresistance in HNSCC cells. (**A**) Representative autoradiogram of EMSA analysis showing complete muting of NFκB DNA binding activity in IR-induced or NFκB overexpressed cells with ΔIκBα. (**B**) Densitometric analysis of NFκB-DNA binding activity showing significant NFκB silencing with ΔIκBα and significant activation with p50/p65 transfection with NFκB over expression vectors, p50 and p65. (**C**) Histograms showing the results of MTT analysis in p50/p65 over-expressed cells treated with EKB-569 (5.0 µg). NFκB over-expression robustly induced SCC-4 cell survival. Conversely, treating NFκB over-expressed cells with EKB-569 completely (P<0.001) inhibited NFκB-induced SCC-4 cell survival. Like-wise, muting NFκB (with ΔIκBα) completely inhibited IR-induced cell survival. (**D**) Histograms showing cell viability in NFκB muted cells exposed to IR or NFκB overexpressed cells treated with EKB-569. Silencing NFκB significantly inhibited IR-induced cell viability. Like-wise, treating NFκB overexpressed cells with EKB-569 (5.0 µg) completely inhibited NFκB-induced cell viability. (**E**) Nuclear morphology with dual staining showing typical yet increased apoptotic characteristics in NFκB muted cells exposed to IR. NFκB overexpressed cells displayed chromatin with organized structures indicating good viability with normal nuclei. However, treatment with EKB-569 (5.0 µg) significantly inflicted chromatin with blebbing, nuclear condensation, and fragmentation in these NFκB overexpressed cells.

To further identify whether targeting IR-induced NFκB orchestrates EKB-569-induced radiosensitization in HNSCC cells, we adopted two approaches. First, we determined whether IR-induced NFκB regulates induced radioprotection in SCC-4 cells. To achieve this we investigated the alterations in cell viability, survival and death after muting IR-induced NFκB. Ecotopic expression of IR-induced NFκB was inhibited by transient transfection of ΔIκBα. Knocking-out IR-induced NFκB was confirmed with EMSA (Figure 5A&B). Compared to vector controls, knocking out IR-induced NFκB with ΔIκBα significantly (P<0.001) conferred IR-inhibited cell survival (Figure 5C), cell viability (Figure 5D) and enhanced IR-induced cell death (evident with bright orange chromatin with blebbing, nuclear condensation, and fragmentation) dictating the role of IR-induced NFκB in radioresistance. Next, to identify that EKB-569 induced radiosensitization occurs at least in part by targeting IR-induced NFκB, p50/p65 over-expressed SCC-4 cells were treated with EKB-569 and analyzed for cell viability, survival and death. EMSA analysis (Figure 5A) confirmed the robust NFκB DNA-binding activity in p50/p65 transfected SCC-4 cells (Figure 5B). Further, over expression of NFκB in these cells significantly (P<0.001) induced cell survival (Figure 5C) and showed bright green chromatin with organized nuclear morphology (Figure 5E) and, served as positive controls for our study. Consequently, treatment with EKB-569 significantly (P<0.001) inhibited cell viability, survival and showed bright orange chromatin with blebbing, nuclear condensation, and fragmentation in these NFκB over-expressed cells (Figure 5C–E) delineating that EKB-569 target NFκB and potentiate cell death in this setting.

## Discussion

Primary and acquired resistance to conventional chemotherapy and radiotherapy represent the central therapeutic challenge in oncology today. Resistance may develop through varied mechanisms, including increased expression of cellular drug efflux pumps; mutation of the therapeutic target; increased activity of DNA repair mechanisms and altered expression of genes involved in apoptotic pathways. To overcome these resistance mechanisms, conventional cancer treatments are increasingly combined with molecularly targeted therapies. Because cytotoxic and targeted therapies have distinct biologic effects and toxicity profiles, such combinations are both rational and well tolerated. To date, the molecular pathway most frequently targeted in combination with conventional chemotherapy or radiotherapy is that of the EGFR. After activation by binding of the EGF and other natural ligands, EGFR activates prosurvival, pro-angiogenic, and anti-apoptotic pathways that may confer resistance to cytotoxic therapies. Interestingly, all these aforementioned functional pathways are known to be controlled by transcriptional master switch regulator, NFκB that also happens to be a downstream target for EGFR. In this study, we investigated the specific inhibitory effect of EGFR TK inhibitor EKB-569 on the regulation of NFκB-dependent survival advantage and elucidated its influence in potentiating radiotherapy for head and neck cancers. To our knowledge, for the first time, we have demonstrated the specific inhibition of IR-induced NFκB with irreversible EGFR TK inhibitor, EKB-569 and dissected out the functional downstream signaling that orchestrate in promoting radiosensitization at least in head neck cancer.

Our results indicate that radiation at clinically relevant doses activated NFκB pathway in SCC-4 cells through the mechanism that interacted with EGFR. To that note, activation of EGFR intrinsic receptor protein TK and tyrosine autophosphorylation results in the activation of a number of key signaling pathways [31]. One major downstream signaling route is via Ras-Raf-MAPK pathway [32] where activation of Ras initiates a multistep phosphorylation cascade that leads to the activation of ERK1 and 2 [33] that regulate transcription of molecules that are linked to cell proliferation, survival, and transformation [33]. Another important target in EGFR signaling is PI3K and the downstream protein-serine/threonine kinase Akt [34], [35] which transduces signals that trigger a cascade of responses from cell growth and proliferation to survival and motility [35]. One more route is via the stress-activated protein kinase pathway, involving protein kinase C and Jak/Stat. Interestingly, the activation of these pathways converges into distinct transcriptional program involving NFκB that mediate cellular responses, including cell division, survival (or death), motility, invasion, adhesion, and cellular repair [25]. QPCR profiling revealed a significant increase in these EGFR dependent NFκB activating molecules viz. *Akt1*, *Jun*, *Map3K1*, *Raf1* after IR and, EKB-569 treatment resulted in complete suppression of these molecules and serve as the positive controls for the study.

Transformed cells have been shown to possess deregulated apoptotic machinery [36]. Transcriptional regulators that regulate pro-apoptotic and/or activate anti-apoptotic proteins play a key role in switching the therapy associated balance of apoptotic cell death. In this regard, EGFR blockers appear to inhibit tumor cell death via multiple mechanisms. EGFR-mediated signaling via the Ras-Raf-MAPK, PI3-K/Akt or PKC-Jak/STAT pathways leads to the activation of NFκB which in turn imbalance the pro/anti-apoptotic protein expression. As is evident from our data, IR-induced NFκB and NFκB-dependent metabolic activity, cell viability and cell death indicate NFκB's direct role in induced radioresistance. Consistently, in multiple tumor cells, we and others have extensively documented that RT induces NFκB activity and delineated its direct role in induced radioresistance [29], [37]–[43]. Conversely, muting NFκB function has been shown to restore apoptosis [44] and confer apoptotic effect in chemo and/or radioresistant tumor cells [45]. Consistently, we observed a complete inhibition of IR-induced NFκB activity with EKB-569 designating that this compound may rectify IR-induced aberrant apoptotic machinery. These results though confirmed that the mechanism of EKB-569-mediated radiosensitization of squamous cell carcinoma is acting specifically through NF-kB pathway, it is interesting to note an induction in the activity of other transcription factors, AP-1 and SP-1. This differential mechanism in the activation of NFκB versus AP-1 and SP-1 may be speculated partly as cell type- and/or stimuli-specific. However, addressing the complete mechanism involved in the induction of IR-induced AP-1 and SP-1 with EKB-569 treatment and its impact on radiosensitization compared to other EGFR-TK inhibitors may help in ascertain the complexity in the combination treatments.

It is also interesting to note form this study that the inhibition of NFκB signaling pathway is not a EKB-569 compound-specific effect. Other commonly used irreversible EGFR blockers, afatinib and neratinib (HKI-272) dose-dependently inhibit NFκB DNA-binding activity. The inhibition of NFκB by these two related compounds was found to be persistent up to at least 72 h as seen with EKB-569 treatment. Similarly, all three EGFR inhibitors, EKB-569, afatinib and neratinib directly inhibit NFκB activity by blocking the activity of IR-induced upstream IκB kinase beta (IKK-β). This direct action of inhibition of NF-kB is EGFR-dependent. EGFR-knockdown experiments with a widely used specific EGFR inhibitor, PD153035 confirmed the EGFR-mediated inhibition of NFκB DNA-binding activity and mRNA expression in the irradiated cells. Therefore the proposed combination of IR and EGFR/NFκB inhibition can be carried out on to the clinic with any EGFR inhibitor compounds other than EKB-569.

To further substantiate our findings, we analyzed the efficacy of EKB-569 in IR-modulated NFκB signaling pathway transcriptional response. Interestingly, EKB-569 robustly modulates the transcriptional response of NFκB signal transduction and downstream mediators of this pathway in SCC-4 cells. To that note, EKB-569 inhibited IR-induced transcription of pro-survival molecules in this setting. Disruption of aberrantly regulated survival signaling mediated by NFκB has recently become an important task in the therapy of several chemoresistant and radioresistant cancers [46]. Anti-apoptotic molecules are expressed at high levels in many tumors and have been reported to contribute to the resistance of cancers to RT [47]. Because activation of caspases plays a central role in the apoptotic machinery [47], therapeutic modulation of molecules such as IAPs could target the core control point that overturn the cell fate and determine sensitivity to RT [48]–[51]. A recent body of evidence has emphasized a central role for NFκB in the control of cell proliferation and survival. NFκB enhances cell survival by switching on the activation of pro-survival molecules that dampen pro-apoptotic signals and attenuate apoptotic response to anticancer drugs and IR [52], [53]. In this perspective, we recently demonstrated that muting IR-induced NFκB regulates NFκB dependent pro-survival molecules and potentiate radiosensitization at least in breast cancer and neuroblastoma models. To our knowledge, the present study for the first time throws light on the efficacy of EKB-569 in regulating IR altered NFκB signal transduction and downstream effector molecules in HNSCC cells. This insight into the comprehensive regulation of IR-induced survival transcription recognizes EKB-569 as “potential radiosensitizer” and further allows us to identify the role of EGFR dependent NFκB mediated orchestration of radioresistance at least in HNSCC.

Though a plethora of studies dissected out the EGFR downstream signaling (some of them discussed above) and suggested that these signaling converge at transcriptional machinery, there remained a paucity of information on the role of specific transcriptional switch in orchestrating EGFR dependent tumor progression. Not only, this study throws light on the molecular blue print that underlies after clinical doses of IR in HNSCC, this study also identifies the potential of the EGFR TK, EKB-569 in selectively targeting IR-induced NFκB and subsequent tumor progression. In this regard, p65 subunit of NFκB is constitutively activated in 70% of HNSCC and IR-induced NFκB plays an important role in HNSCC resistance to RT. Though constitutive and RT-induced NFκB has been causally linked to induced-radioresistance, its precise participation in RT-induced cell death orchestration is poorly understood. In this regard, results of the present study exhibit that ecotopically muting IR-induced NFκB with ΔIκBα robustly induced cell death in HNSCC cells demonstrating that IR-induced NFκB regulates cell death at least in this setting. Furthermore, to causally delineate that EKB-569 dependent silencing of NFκB mediates the induced radiosensitization, we analyzed their effect on NFκB overexpressed cells. For the first time, the results of the present study imply that EKB-569 inhibits HNSCC cell survival and viability by selectively targeting NFκB.

In summary, these results demonstrate that EKB-569 significantly inhibits IR-induced NFκB activity in human HNSCC cells. Furthermore, this study identifies the EKB-569-associated inhibition of NFκB pathway survival signaling blue print, more precisely to the regimen of the treatment modality, in this case IR. Evidently, treatment with EKB-569 profoundly conferred IR-inhibited HNSCC cell survival and viability. Consistently, this EGFR TK significantly enhanced IR-induced HNSCC apoptosis. More importantly, NFκB over expression and knockout studies demonstrated that EKB-569-associated targeting of IR-induced NFκB mediates cell death in HNSCC cells. Taken together, these data strongly suggest that EKB-569 may exert radiosensitization at least in part by selectively targeting IR-induced NFκB dependent survival signaling, that potentiate radiotherapy in effective HNSCC cell killing. Further in-depth *in vivo* studies are warranted to verify this suggestion and are presently under investigation in our laboratory.

## Supporting Information

Figure S1 **QPCR profiling amplification charts and heat map showing transcriptional changes in 88 NFκB-dependent downstream target genes in SCC-4 cells.** Cells were either mock-irradiated, exposed to IR or pretreated with EKB-569 (5 ug) and then exposed to IR. Real-time QPCR profiling was performed using human NFκB signaling pathway profiler (Realtimeprimers.com, Elkins Park, PA).
(TIF)
