# Supplementary figures and images for: Irreversible EGFR Inhibitor EKB-569 Targets Low-LET γ-Radiation-Triggered Rel Orchestration and Potentiates Cell Death in Squamous Cell Carcinoma
Source: PLoS One. 2011 Dec 29;6(12):e29705. doi: 10.1371/journal.pone.0029705 (PMC3248439; doi:10.1371/journal.pone.0029705)

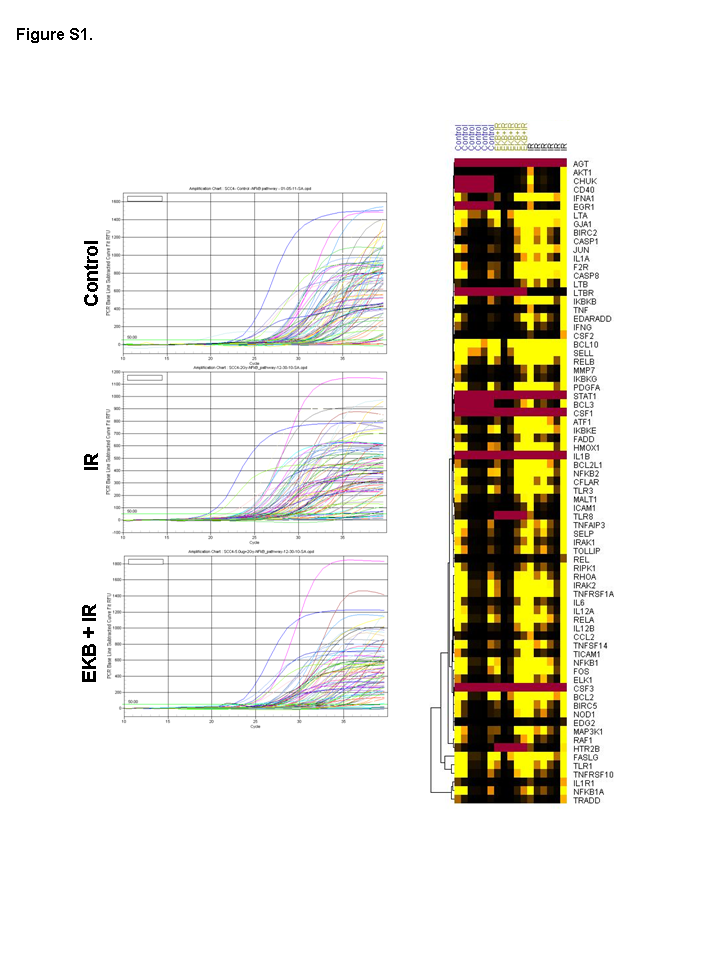

Supplement: Figure S1 — QPCR profiling amplification charts and heat map showing transcriptional changes in 88 NFκB-dependent downstream target genes in SCC-4 cells. Cells were either mock-irradiated, exposed to IR or pretreated with EKB-569 (5 ug) and then exposed to IR. Real-time QPCR profiling was performed using human NFκB signaling pathway profiler (Realtimeprimers.com, Elkins Park, PA). (TIF) [file pone.0029705.s001.tif]
